# Possible etiologies of restless legs syndrome in pregnancy: a narrative review

**DOI:** 10.5935/1984-0063.20220080

**Published:** 2022

**Authors:** Ana Mendes, Vitória Silva

**Affiliations:** Centro Hospitalar De Trás-Os-Montes E Alto Douro, E.P.E., Pulmonology - Vila Real - Portugal

**Keywords:** Restless Legs Syndrome, Pregnancy, Hormones, Elements, Vitamin D, Genetics

## Abstract

Restless legs syndrome (RLS) is a sensorimotor disorder characterized by an urgent need to move the legs, due to the presence of a discomfort sensation in the lower limbs, especially at rest. Generally, it relieves with movement. There are several studies that argue the existence of an association between this syndrome and pregnancy. However, the pathophysiological mechanisms of this disorder in pregnancy are misunderstood. The objective of this narrative review is to identify and discuss some possible etiologies of RLS in pregnancy. A literature search was performed in the PubMed and ResearchGate databases by using the following search strategies: “restless legs syndrome”, “restless legs syndrome in pregnancy”, “pregnancy and vitamin D deficiency” and “pregnancy and zinc”. The publications were initially sorted through their title. After the initial process, inclusion and exclusion criteria were applied. The included articles were sorted by authors, year, journal of publication, type of study, and organized by chronological order of publication. Among the main findings, hormonal changes, iron metabolism, vitamin D deficiency, genetic factors, zinc and magnesium fluctuations have been some of the hypotheses supporting the development or worsening of this disorder in pregnancy. Dopamine also appears to be correlated with hormonal changes, iron metabolism, ferritin, folic acid and vitamin D deficiency. In conclusion, there are several hypotheses trying to link restless legs syndrome with pregnancy. The most covered were hormonal fluctuations and iron metabolism. However, this thematic is still highly discussed, creating the need for additional and thorough research.

## INTRODUCTION

Restless legs syndrome (RLS) also known as Willis-Ekbom’s disease is a sensorimotor disorder, characterized by an urgent need to move the legs, due to a sensation of discomfort in the lower limbs^1-3^. Usually the symptoms occur in inactivity periods and relieve with movement^1,2^.

The prevalence of this syndrome in pregnancy varies with the gestational week, being the third trimester the period with the highest values^1,3-5^. After delivery, its tendency is to decrease^4-6^. The risk of developing chronic RLS is fourfold superior when transient RLS is present during gestation^7^. In some cases, the idiopathic form of this illness may develop if symptoms appear for the first time during pregnancy and persist after delivery^6^.

According to the meta-analysis by Chen et al. (2018)^8^, in the first trimester of pregnancy, the prevalence of this disease reaches 8%, obtaining higher values in the second and third trimesters: 16% and 22%, respectively. After delivery, it decreases to 4%. Agreeing to this article, the prevalence of this disorder in pregnancy presents some geographical variability. Thus, it is more prevalent in the Eastern Mediterranean region, with values of 30%. In Europe and America regions, the prevalence is 22% and 20%, respectively. In the Western Pacific Region, it is lower, reaching 14%^8^.

This illness can be classified according to its etiology into primary and secondary^9,10^. In its primary form most cases can have an associated genetic component^9^. The secondary form can be related to further conditions, such as iron deficiency, pregnancy, chronic kidney disease, among others^9,10^.

This disease can have a negative impact on life and may also cause adverse effects on cognitive function and sleep quality. In pregnant women, it can develop complications in childbirth and fetal growth^11^. An early diagnosis is important and must be done through a detailed anamnesis, based on the criteria proposed by the *International Restless Leg Syndrome Study Group* (IRLSSG)^6,7^. Adequate treatment is essential to prevent complications and health problems^6^.

There are several hypotheses trying to establish an association between RLS and gestation. However, the pathophysiological mechanisms of this disorder in pregnancy are misunderstood and further investigations are required^1,12,13^. Hormonal changes, the metabolism of iron, ferritin and folate, vitamin D deficiency, genetic factors and variations in zinc and magnesium levels have been hypothesized^1,6,10,14^.

This thematic is still highly discussed, so it is relevant to elaborate a narrative review. The main objective of this narrative review is to identify and discuss possible etiologies of RLS in pregnancy.

## BIBLIOGRAPHIC SEARCH

A literature search was performed in the PubMed and ResearchGate by using the following search strategies: “restless legs syndrome”, “restless legs syndrome in pregnancy”, “pregnancy and vitamin D deficiency” and “pregnancy and zinc”. The search was also performed by using the corresponding terms in Portuguese and Spanish.

The research period began in July 2020 and the latest database search was performed in February 2021.

Initially the publications founded in databases were selected through their title and abstract.

The inclusion criteria were: scientific articles with results and conclusions about possible etiologies of restless leg syndrome in pregnancy; literature with relevant concepts to an understanding of the thematic and/or that described the pathophysiological mechanisms of this disease, preferably in pregnancy; literature written in English, Portuguese and Spanish.

The exclusion criteria were: articles under the year 2000; literature that did not contain relevant information for the theme under analysis, as the potential association between gestational RLS with other health conditions, such as renal disease, obstructive sleep apnea hypopnea syndrome, diabetes mellitus, obesity, and preeclampsia.

The included articles were sorted by authors, year, journal of publication, and type of study. The full reading and analysis of the included studies also offered the possibility to access other publications, which made it possible to include 29 references.

**Figure 1:**
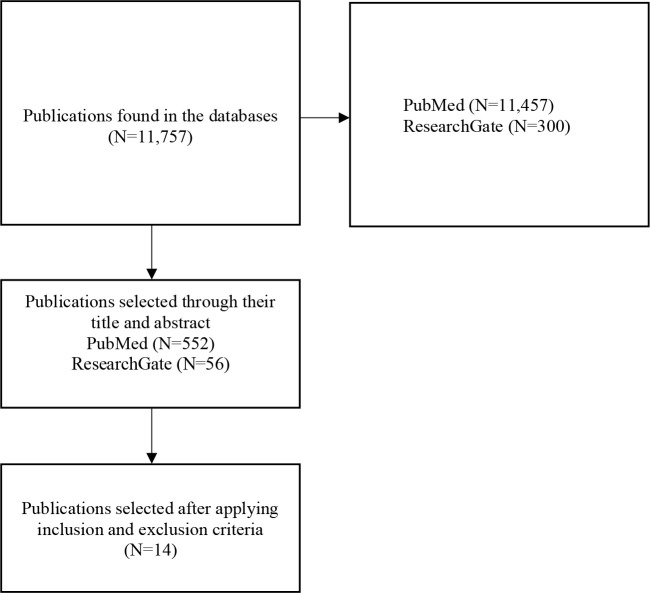
Flowchart of the publications selection process.

The process of selecting publications in the databases according to search strategies were shown in the flowchart presented in Figure 1.

After applying the inclusion and exclusion criteria, 14 publications were selected. The publications are presented in Table 1 by chronological order of publication.

**Table 1:** Articles organized by authors, year, journal of publication, and type of study.

Publications	Authors	Year	Journal of publication	Type of study
1 ^23^	Pereira Junior et al.	2010	Clinics	Review
2 ^10^	Srivanitchapoom et al.	2014	Parkinsonism & Related Disorders	Review
3 ^16^	Shang et al.	2015	Sleep and Breathing	Cross-sectional
4 ^26^	Neyal et al.	2015	Sleep Medicine	Prospective Longitudinal
5 ^12^	Prosperetti and Manconi	2015	Sleep Medicine Clinics	Review
6 ^21^	Grover et al.	2015	Obstetric Medicine	Review
7 ^6^	Gupta R et al.	2016	Acta Neurologica Scandinavica	Review
8 ^8^	Chen et al.	2018	Sleep Medicine Reviews	Systematic review and meta-analysis
9 ^1^	Garbazza and Manconi	2018	Sleep Medicine Clinics	Review
10 ^17^	Seeman	2020	International Journal of Environmental Research and Public Health	Review
11 ^25^	Almeneessie et al.	2020	Annals of Thoracic Medicine	Case-control
12 ^33^	Sağlam et al.	2020	Journal Of Turkish Sleep Medicine	Cross-sectional
13 ^3^	Yıldırım and Apaydın	2020	Biological Trace Element Research	Case-control
14 ^2^	Cimsir and Savas	2021	Journal of Clinical and Experimental Investigations	Randomized controlled

## DISCUSSION

The present narrative review arises from the lack of consensus and divergent views about the topic. It is intended to identify some studies that describe our problem of interest: *“Possible Etiologies of Restless Legs Syndrome in Pregnancy”*. It is not planned to present new data, only assess what is already published, summarizing and trying to provide the best currently available evidence, avoiding duplications, and seeking new study areas not yet addressed.

### Hormones

The presence of hormonal changes during gestation, especially those related to the levels of estrogen, progesterone, prolactin, and thyroid hormones have been hypotheses that support the development of RLS in pregnancy^1,7,10,15,16^.

The level of estrogen, despite being considered a possible explanation for this pathology in pregnancy, is still an underdeveloped issue.

There is insufficient evidence about how this syndrome can be triggered or exacerbated by estrogen^10^. However, it may be connected to the interaction between estrogen and dopamine^17^. According to Seeman (2020)^17^, estrogen possibly acts as a dopamine antagonist in RLS, similarly to what happens in schizophrenia. Supporting this hypothesis, Chen et al. (2018)^8^, by mentioning other studies, defends the idea that estrogen can affect dopamine synthesis and release, inhibiting it^8,16^. When there is a compromise of dopamine flow into the bloodstream of the anterior pituitary gland, there may be an ineffective lactotroph suppression, potentiating the hypersecretion of prolactin, another hormone that seems to be related to this syndrome^10^.

Dopamine is involved in innumerous processes, among which motor control functions. Thus, the presence of a dopaminergic imbalance or dysfunction in the nigrostriatal system may contribute to the development or worsening of this disorder^10^.

There is evidence that estradiol levels and pregnancy-related RLS may be correlated^3^. Their prevalence is higher in the third trimester. After delivery, a decline in estradiol levels occurs, as well as the prevalence and severity of the syndrome^10^. The study by Dzaja et al. (2009)^18^ had as main objective to clarify the relationship between hormonal and metabolic changes during pregnancy and in the postpartum period with RLS symptoms. Thus, blood samples were drawn from 29 pregnant women: 10 diagnosed with RLS (mean age ± SD, 31.6 ± 2.4y) and 9 healthy (control group, mean age ± SD, 32.9 ± 2.7y). In the RLS group, 8 mentioned the presence of RLS symptoms prior to the current pregnancy. All described worsening of symptoms with pregnancy. Comparing the results of the two study groups (RLS vs. controls), it was found that estradiol levels were higher during pregnancy in both groups. However, it was more marked in RLS group during pregnancy, regardless of the new-onset or preexisting RLS symptoms (estradiol_RLS_: 34.211 ± 6,397pg/mL vs. estradiol_controls_: 25.475 ± 7.990pg/mL, *p*<0.05)^10,18^.

Similar to this study, Tunç et al. (2007)^19^ analyzed a group of pregnant women (n=146) with the mean age around 24.81 ± 5.01 years. The principal aim was to identify risk factors for RLS in this type of sample. Among the participants, 38 (26.02%) were diagnosed with RLS. It was performed in all participants (RLS^+^ vs. RLS^-^) routine blood biochemistry tests, complete blood count and thyroid functions tests. The laboratory values demonstrated that in both groups the estradiol levels were similar (estradiol_RLS+_ = 4,187. 2 ± 469.9pg/mL vs. estradiol_RLS-_ = 4,193.26 ± 435.46pg/mL), revealing that there was no significant difference between estradiol levels in pregnant women with the disease and those without (*p*=0.916)^19^. Referring to the cohort study of Hübner et al. (2013)^20^, its aim was to evaluate characteristics and determinants of RLS in gestation and its impact on sleep quality. The estrogen levels in the third trimester were measured in 15 patients with RLS and in 20 patients without the disease. In the group of patients with RLS, it was observed a lower value in estrogen levels (mean 57,865 ± 13,578pg/mL) compared with the group without RLS (mean 63,900 ± 18,923pg/mL). However, there was also no considerable difference in estrogen levels between the women studied.

Progesterone, another essential hormone in pregnancy, increases during gestation, reaching its highest values in the third trimester^10^. This hormone seems to have the function of raising the sensitivity of the respiratory center to carbon dioxide and increasing neuronal excitability^19,21^. This hyperexcitability has been a hypothesis to an explanation for the development of RLS^19,21^. According to Srivanitchapoom et al. (2014)^10^, there is an interaction in the striatum between progesterone and dopamine. However, the exact mechanism between the two is still unknown.

The influence of prolactin on the pathophysiology of RLS during pregnancy has also been analyzed. According to Garcia-Borreguero et al. (2004)^22^, there is apparently a correlation between periodic leg movements and plasma prolactin levels^8^. Interestingly, the symptoms of the disease have the same circadian rhythmicity as prolactin^21^. Agreeing to Grover et al. (2015)^21^, this hormone, when secreted during gestation, can decrease the action of dopamine. This reduction may explain the worsening of symptoms^21^. However, there is also evidence that, after delivery, most women with this disorder reveal a symptomology improvement, while prolactin secretion continues to increase^8^.

Thyroid hormones levels alterations can be present during pregnancy, seeming to be correlated to the expression of this disorder. Nevertheless, this is still a hypothesis under discussion.

The disease and its symptoms may be triggered by elevated levels of these hormones during gestation and by hyperthyroidism^6,23^. Agreeing to this theory, this syndrome may be induced by deficient dopamine production and decreased catabolism of thyroxine, both arising from iron deficiency in pregnancy^6,23^. This illness can then develop from an imbalance between the elevation of thyroid hormones and the dopaminergic agonists’ system^23^. Nonetheless, the absolute values of thyroxine and thyrotropin in gestation may not accurately reflect the thyroid status, and their optimal values are still unknown in this gestational context^6^.

According to Pereira et al. (2010)^23^, the augmented levels of thyroid hormones can be explained by the elevation of estradiol during pregnancy.

The role of thyroid hormones in the manifestation of this disease during gestation has been investigated over time. In the study by Cimsir and Savas (2021)^22^, the main objective was to determine the prevalence of RLS during gestation and possible factors affecting its etiology. To achieve this goal, the sample included 99 pregnant women and was divided into two different groups: those with RLS (n=31, mean age: 29.2 ± 5.9y) and those without (n=68, mean age: 29.57 ± 6.09y). Comparing to the group without the disorder, higher values of thyroid stimulating hormone were found in the pregnant women with RLS (1.97 ± 1.34µU/ml vs. 2.09 ± 1.14µU/ml, respectively). However, there was no significant difference in the values of this hormone between the groups (*p*=0.660)^2^. Also in Çakmak’s article, with an identical aim as Cimsir and Savas (2021)^22^, there was no significant difference in thyroid hormone levels or history of thyroid disease between the pregnant women with and without the syndrome (*p*>0.05)^24^. In this study, 500 pregnant women with a mean age of 27.0 ± 5.9 years were evaluated and were divided into two groups: RLS (n=77) and non-RLS (n=423) groups. The incidence of RLS in pregnancy was 15.4%. The thyroid-stimulating hormone and thyroxine values in the RLS group were 2.3 ± 1.4mlU/mL and 1.0 ± 0.2ng/mL, respectively. In the non-RLS group, the values were similar to the RLS group (1.8 ± 1.4mlU/mL; 1.8 ± 0.2ng/mL, respectively). The results about thyroid disease were not in agreement with the study by Shang et al. (2015)^16^. One of the main goals of this study was to explore potential factors of RLS and its severity during different trimesters. To achieve this goal, 1,584 pregnant Chinese women (mean age: 26.0 ± 6.4 years) were part of the sample. Only 177 participants develop RLS during pregnancy. Comparing some results between RLS and non-RLS groups, a higher prevalence of thyroid disorders was demonstrated in pregnant women with RLS (5.6%) versus those with the absence of the disorder (2.4%) (*p*=0.014)^16^.

Increased levels of estrogen, progesterone, prolactin, and thyroid hormones seem to correlate with dopamine.

Although hormonal changes may be a possible explanation for RLS in pregnancy, there are controversies, once this syndrome occurs in less than a third of patients^10^. In order to make this issue more conclusive, and regarding the scarcity of current articles, additional studies are necessary.

### Iron, ferritin and folate

During gestation, iron, ferritin, and serum folate levels decrease, as well as hemoglobin, another iron indicator^1,10^. These changes can be explained by an increase of total blood volume, resulting in a dilution of these components and by the fetus’ augmented needs, whose development depends on iron and folate^1,10,12^. There is evidence that the number of pregnancies can influence iron levels. If not restored between gestations, its tendency is to decrease with new pregnancies. So, multiparity appears to be associated with an increased risk of developing RLS^6,25^.

Over time, investigations have demonstrated the involvement of iron and tetrahydrobiopterin in the dopaminergic system, acting as co-factors of the enzyme tyrosine hydroxylase, which has a relevant part in dopamine production^10,15,26^. Folic acid plays a key role in the regeneration of tetrahydrobiopterin^10^. Thus, if there is a decrease in iron and folic acid, dopamine synthesis may be limited, influencing the pathogenesis of the disease^1,10^.

The symptoms of this disorder in pregnant women may be aggravated due to an imbalance in iron metabolism, transport and storage^26^. However, this is insufficiently clarified in pregnancy.

The role of iron on the pathogenesis of this syndrome has been investigated. Ferré et al. (2018)^27^ refers that iron deficiency may also influence the glutamatergic system. According to Seeman (2020)^17^, reporting Jiménez-Jiménez et al. (2019)^28^, glutamate, gamma-hydroxybutyric acid and adenosine seems to play an important part in the etiology of RLS^17,28^. As described by Ferré et al. (2018)^27^, in the presence of normal iron levels in the brain region, extracellular concentrations of adenosine, mediated by A1 receptors, maintains an inhibitory presynaptic tone on glutamatergic and dopaminergic terminals in the striatum. An iron deficiency condition leads to downregulation of A1 receptors, causing a hypersensitivity of the glutamatergic and dopaminergic terminals. Thus, a hyperglutamatergic and hyperdopaminergic state is generated, which may be a sufficient pathophysiological mechanism to explain the periodic leg movements associated with this syndrome^27^.

Referring Telarović et al. (2019)^29^ RLS appears more commonly in anemic pregnant women. To reach this conclusion, this study was developed on a sample of 462 women aged 18-50 years and 231 of them were pregnant. One of the research objectives was to compare in pregnant women with RLS and those without, the frequency of iron deficiency anemia. The results demonstrated the presence of this illness in 17.6% of pregnant women who had no signs of anemia and in 38.6% of anemic women. The statistical analysis methods used demonstrated that this difference was considered statistically significant (t=2.67, *p*=0.008). In the study by Minár et al. (2015)^30^, the principal aim was to determine possible risk factors for developing RLS in pregnancy. This study admitted 300 pregnant women in the last trimester with a mean age of 30.81 years, however only 94 (31.33%) fulfilled the criteria for RLS. Blood samples were collected and some parameters of iron metabolism (levels of hemoglobin and hematocrit, ferritin, mean corpuscular volume, mean corpuscular hemoglobin and mean corpuscular hemoglobin concentration) were examined.

Among the results, the decrease in hemoglobin levels, mean corpuscular hemoglobin and mean corpuscular hemoglobin concentration was significantly evidenced in pregnant women with the syndrome, demonstrating the presence of iron deficiency (*p*<0.05)^30^. In the same group, was observed the appearance or worsening of symptoms, in the third trimester, a period of higher iron consumption by the fetus^30^. In the study by Sikandar et al. (2009)^31^, 271 pregnant women of Pakistan were admitted and 30% of them were diagnosed with RLS. The objective was to determine the frequency of RLS in pregnant women and its predictors in gestation. One of the variables under investigation was serum hemoglobin level ≤11g/dL and it was considered an indicator of iron deficiency anemia^31^. Comparing the group with RLS (n=81) to the group without the disease (n=190), it was possible to conclude that it might be an independent predictor for this syndrome during pregnancy (*p-value*<0.036)^31^. However, it was also evident that this explanation cannot be individually considered, due to an existing controversy about the effects of iron supplementation in disease improvement^31^.

In order to determine a possible association between mean folic acid levels and RLS in gestation, Morker et al. (2017)^32^ conducted a study with a sample of 107 women (aged ≥18 years). However, due to a lab error, the folic acid levels in 8 women were not disposable. Hence, the sample was reassessed (n=99). In 99 women, 20 were in the gestation RLS group and 79 were included in the group with no disease. Comparing mean folic acid levels in these two groups, it was possible to verify a significant difference in its levels (*p*>0.05), between pregnant women with RLS (27.3 ± 12.9ng/mL) and the group without (32.2 ± 20.6ng/mL). According to this study, folic acid supplementation can be implemented in gestation where moderate to severe RLS is present^32^.

Garbazza and Manconi (2018)^1^ argue that a decrease in serum ferritin, in the early stage of pregnancy or preceding it, has been a predictor of RLS throughout gestation.

The main objective of the longitudinal prospective study by Neyal et al. (2015)^26^ was to investigate the correlates of WED/RLS during and after pregnancy. This investigation included a total of 389 pregnant women: 138 with RLS (mean age: 27.8 ± 5.8 years) and 251 (mean age: 27.9 ± 6.0 years) without the disease. Some laboratory investigations were realized and the results demonstrated a significant decrease in ferritin levels (*p*=0.010), transferrin saturation (*p*=0.004), and blood urea nitrogen (*p*=0.040) was observed in the group of gestating women with the illness^5,26^. There was verified an association between decreased ferritin levels during pregnancy and symptoms of the disorder after delivery^26^. Interestingly, Minár et al. (2015)^30^ establishes that serum ferritin levels did not correlate with the severity of this syndrome, finding no significant differences in ferritin levels in pregnant women, with and without the disease. Also in the study by Cimsir and Savas (2021)^22^, despite the decrease in ferritin levels in pregnant women with the disorder (n=31), there was no significant difference between both groups analyzed (*p*=0.413)^2^.

The influence of iron and folate on the pathogenesis of this syndrome in pregnancy remains doubtful. This evidence is supported by a rapidly decrease of the symptoms of RLS, after delivery, whereas iron and folate levels are gradually restored, and also due to the ineffectiveness of oral folate and iron supplementation in preventing the symptoms^1,8,10,16^. The divergence in the findings of various studies require further investigations.

### Vitamin D

Vitamin D has an important role in the regulation of calcium levels in the brain, in neuroprotection and neuromodulation, as well as in iron and dopamine metabolism^33,34^. The effect of this vitamin on various neurological conditions has been analyzed, after the discovery of its receptors in the thalamus, hypothalamus, substantia nigra and cortex^34^. This vitamin has a protective effect on dopaminergic neurons against toxins and it is responsible for the increase of dopamine levels in the brain^33,35^.

Throughout pregnancy, a decrease of vitamin D is common^6^. It is known that its deficiency facilitates the appearance of several complications, such as preeclampsia, gestational diabetes, prematurity and low weight newborns. This deficiency, evidenced during pregnancy, may also have harmful consequences in children under 5 years of age, as the development of asthma^36^.

According to a study elaborated by Gür et al. (2014)^36^, it was possible to evaluate the prevalence and risk factors for vitamin D deficiency in mothers and healthy newborns. Blood samples were realized and laboratory studies were performed. For mothers, vitamin D levels were categorized in three groups: group I (vitamin D deficient) for serum 25 (OH) D3 ≤ 20ng/mL, group II (vitamin D insufficient) for serum 25 (OH) D3 = 21-29ng/mL and group III (normal vitamin D) for serum 25 (OH) D3 ≥ 30ng/mL. In healthy pregnant women, 62.6% had a vitamin D deficiency and 18.2% had its insufficiency^36^. In agreement with this research is Al-Faris’ (2016)^37^ study. The vitamin D status was divided in the following categories: deficient (25(OH) D < 50nmol/L), insufficient (25(OH) D = 50-74nmol/L) and sufficient (25(OH) D ≥ 75nmol/L). In a sample of 160 Saudi pregnant women aged between 20-34 years, the investigation revealed a vitamin D deficiency in 50% and its insufficiency in 43.8%^37^.

A deficit of this vitamin may interfere with dopaminergic neurotransmission and it can contribute to an imbalance in dopamine levels. Therefore, its involvement has been linked to the pathogenesis of RLS in pregnancy^6,33^.

Referring Sağlam et al. (2020)^33^, vitamin D is related to the severity of RLS in pregnant women. This investigation was conducted in a sample of 145 pregnant women and had as main goal the association between vitamin D deficiency and the prevalence and severity of RLS. The sample was divided in two groups: group 1 (mean age: 27.0 ± 6.1) with low 25 (OH) vitamin D (<20ng/mL) and group 2 (mean age: 28.1 ± 5.1) with normal 25 (OH) vitamin D levels (≥20ng/mL)^33^.

The results reveal in 70 pregnant women the presence of RLS. With the disease, 57 (58.2%) were in group 1 and 13 (27.7%) were included in group 2. Other statistical analysis was performed and revealed that RLS severity was significantly higher in group 1 (*p*=0.001). In the presence of lower plasma concentrations of 25 (OH) vitamin D, and using the severity scale of the IRLSSG, it is expected to develop a severest form of this syndrome^33^.

Almeneessie et al. (2020)^25^ investigated the prevalence of RLS, its correlates and severity among Saudi pregnant women. The sample included 742 pregnant women and 742 non-pregnant women. Among pregnant women, RLS was absent in 519 (mean age: 28.8 ± 5.3) and present in 223 (mean age: 30.1 ± 5.9). The vitamin D deficiency was also evaluated. Comparing the group with RLS vs. those without, it was higher in pregnant women with RLS (21%) and the difference was statistically significant (*p*=0.005). So, among other factors, they settled that the deficiency of this vitamin can be associated with the etiology of this disorder. However, this study presented some limitations in its methodology^25^.

The pathophysiological role of this vitamin in RLS has been supported by the presence of augmented concentrations of vitamin D binding protein in the cerebrospinal fluid of patients with this syndrome^6,25^. However, its usage for the treatment of this disorder if still doubtful and there is no evidence of symptomatology improvement^25^. The deficit of this vitamin as a trigger factor of this disorder in pregnancy is still a controversial topic. Thus, it is pertinent to perform complementary studies.

### Genetic factors

Several studies have supported the theory that RLS in gestation may be associated with genetic factors^6,20^.

It is suspected that in women genetically predisposed to the disorder, pregnancy may trigger its symptomatology^1,6^. The symptoms exacerbate during gestation, in most women with a preexisting form of this illness^1^. Women who had previously experienced RLS, when pregnant, have a higher risk of symptoms reappearance in future pregnancies^6,12^.

Familial RLS is much more common in pregnant women with the disorder than in women with its secondary forms or without it^1^. The risk of developing RLS in its idiopathic form is 3 to 4-fold augmented in women who have had the illness during gestation^12^.

The investigation by Cesnik et al. (2010)^38^, was one of the studies with the purpose to study if pregnancy-related RLS in its transitory form might be considered an important risk factor for developing a future chronic RLS form. In this study, 74 women with pregnancy-related RLS (mean age 35.15 ± 5 years) and 133 who have never experienced RLS (control group - mean age 37.85 ± 4 years), were included. During the follow-up time (6 years), 28 women were diagnosed with RLS: 18 belonging to the pregnancy-related RLS group and 10 in the control group, corresponding to a prevalence of 24.3% and 7.5%, respectively^38^.

In these 6 years, 57 women were pregnant again: 33 from the control group and 24 from the pregnancy-related RLS group. The symptoms of RLS occur in only one woman of the control group (3%) and in 14 women of the pregnancy-related RLS group (58.3%). Thus, this investigation concluded that transient RLS in pregnancy was considered an important risk factor for the manifestation of a future chronic idiopathic form of the disorder as well as a new transition RLS form in future gestations^38^.

The incidence per 1.000 person-years of RLS among women included in the pregnancy-related RLS group (56 per 1.000) was four-fold superior when compared to the incidence in the control group (12.6 per 1.000). The incidence of RLS in its chronic form was also analyzed. It was 3 times higher in the group of women with RLS related to pregnancy (34.4 per 1.000) when compared to the control group (11.5 per 1.000)^38^.

The results also demonstrated that the risk in developing RLS was different among the women belonging to the pregnancy-related RLS group. Some of them only experienced the manifestations of RLS during the first pregnancy, others presented RLS symptoms in further pregnancies and the remaining developed RLS in its chronic form^38^.

In a previous investigation (n=606 women; mean age: 31.8 ± 4.7 years) cited in this article and conducted by the same authors, it was hypothesized that pregnancy itself might play an important role in decreasing the threshold risk for RLS in all women, but could also induce RLS symptoms in predisposed women. This study divided the sample in two different groups: pregnancy-related RLS group and those without the illness (control group). The hypothesis proposed was supported by the higher positive family history for RLS present in the pregnancy-related RLS group when compared to the control group. Thus, according to these authors, the genetic background might be a key factor^38^.

Considering that not all pregnant women develop RLS during pregnancy, and the same occurs in other secondary RLS forms, it seems probable that the predisposition to develop RLS in the idiopathic form and in some of the symptomatic ones might be influenced by the genetic background, which appears to play a decisive role^38^.

Recent investigations in the genetic field established that some allelic variants in specific genomic regions might be a clear risk factor in the evolution of RLS. The “Genome-Wide Association Study” recognized risky alleles for the idiopathic form for this syndrome in five specific genomic regions: MEIS1, BTBD9, PTPRD, MAP2k/SKOR1 and TOX3/BC034767, and also in an intergenic region on chromosome 2 (rs6747972)^17^.

Despite some allelic variants predisposing to idiopathic RLS (loci: MEIS1, BTBD9, and MAP2K5) are already known, future investigations focused on the frequency of this variants should be reproduced in a large scale of women with this supposedly “secondary” pregnancy-related RLS form^38^.

Under this context, although pregnancy might be considered an important risk factor, it is still necessary to discover and connect to gestation, a specific genetic predisposition capable of triggering the phenotype of this disease^38^.

Numerous investigations tried to establish an association between this syndrome during pregnancy and the presence of family history. Neyal et al. (2015)^26^ verified that a family history of this illness was present in 8.7% of women diagnosed with RLS. In this study, only 2.5% of the women without the disease had a family history of RLS (p=0.006). However, the symptoms of this disease, after delivery, demonstrate no significant association with the presence of family history of the disease^26^.

In the study by Panvatvanich et al. (2019)^11^, directed in 214 Thai pregnant women (mean age 28.60 ± 6.52 years), the principal objective was to estimate the prevalence, natural course and predictive factors of RLS in this women population. The sample was divided into a group of pregnant women with RLS during gestation (n=24) and a group without it (n=190). Among the parameters analyzed, the presence of a family history of RLS in these women was under discussion. Comparing both groups, 12.5% of the women with RLS during pregnancy had a family history of the disease and only 0.5% of the women in the group without the illness had the same family history (*p*<0.01). Thus, in this investigation it was possible to conclude that a previous history of RLS might be considered a predictor for the appearance of this disease during pregnancy^11^. Still, further genetic studies are required in women with pregnancy-related RLS^1,12^.

### Zinc and Magnesium

Pregnancy is considered a period of intense alterations for a woman. During it, oscillations in zinc and magnesium levels can be observed. Homeostatic changes during gestation can result in zinc deficiency^39^. Several studies have shown that low zinc levels are closely related to the possibility of complications in pregnancy, as well as associated with prematurity and low birth weight^3,39^. A correlation between diminished zinc levels and preeclampsia has also been described^3^. Magnesium has an essential function during pregnancy and in fetal growth. Its deficiency during gestation may occur^40^. Thus, zinc and magnesium have a fundamental role in pregnancy and embryonic development^3^.

According to Yıldırım and Apaydın (2020)^3^ there is a connection between these two elements and RLS. The principal objective was to assess the relationship of RLS that occurs for the first time in pregnancy with clinical and psychiatric data. This investigation was performed in 253 pregnant women and the sample was divided in two groups: healthy pregnant women (n=134, mean age: 27.25 ± 6.89) and pregnant women with RLS (n=119, mean age: 28.10 ± 5.79). Some laboratory analyses were realized in both groups and it was possible to conclude that women diagnosed with this disorder during pregnancy, had lower levels of zinc and magnesium when compared to the healthy group^3^. The study revealed that the difference between groups for each element was statistically significant (*p*<0.001 in both cases). Zinc deficiency can be explained by poor nutritional variety, lack of animal protein intake and caffeine consumption^3^. This study corroborates and mentions the studies of Abdelhaleim et al. (2019)^41^ and Kelkitli et al. (2016)^42^ which, although in non-pregnant participants, investigated the association between iron-deficiency anemia and zinc deficiency with RLS. The first, analyzed 60 participants, and the sample was divided into 30 healthy individuals (mean age: 32.3 ± 5.9) and 30 with iron deficiency anemia (mean age: 31 ± 7.67). Analyzing the biochemical findings, the zinc levels were lower in patients with iron deficiency anemia when compared with the control group (43.4 ± 7.9mg/dL vs. 94.7 ± 16.75mg/dL, respectively). The difference was statistically significant between the groups (*p*<0.0001). In this study, patients with decreased iron and zinc levels had some symptoms including manifestations of RLS^3,41^. The second, evaluated 86 participants, of which 43 were adults with iron deficiency anemia (mean age: 34.95 ± 14.9) and the other 43 were considered the control group (healthy individuals-mean age: 32.05 ± 10.8). In this investigation, a relationship between RLS, iron deficiency anemia and zinc deficiency was demonstrated (*p*=0.016). Investigating simultaneously iron-deficiency anemia and zinc levels, it was found that the illness was diagnosed in 28% of patients with decreased zinc levels^3,42^.

However, the decreased iron present in both studies participants may have bewildered the results, since the deficiency of this element has been presented as a possible explanation to the pathophysiology of RLS^3^.

Yıldırım and Apaydın (2020)^3^ demonstrated that women with pregnancy-related RLS had lower magnesium levels when compared to the healthy group (1.98 ± 0.30mg/dl vs. 2.09 ± 0.21mg/dl, respectively). No additional literature has been found to complement this research. Although, there are investigations pointing out a possible association between magnesium and RLS in non-pregnant participants^3^. One of these studied a sample of 1,107 participants and concluded in this investigation that lower serum magnesium levels increase periodic leg movements during sleep. Likewise, this syndrome was diagnosed in some participants with leg movements^3,43^.

## CONCLUSION

Restless legs syndrome in pregnancy is characterized by the urgent need to move the legs, due to the presence of a discomfort sensation in the lower limbs, mainly when resting. The prevalence of this disorder is higher in the third trimester. The etiology of RLS in gestation is not completely known. However, hormonal fluctuations, iron, ferritin and folate metabolism, vitamin D deficiency, genetic factors, zinc and magnesium changes have been some of the hypotheses that support the development or worsening of this disorder in pregnancy.

Hormonal changes during gestation such as increased levels of estrogen, progesterone, prolactin, and thyroid hormones seem to have a strong link with dopamine, emphasizing the development or worsening of RLS in pregnancy.

Estrogen possibly inhibits dopamine synthesis and release, acting as a dopamine antagonist in RLS. Progesterone appears to increase the sensitivity of the respiratory center to carbon dioxide and increase neuronal excitability. Prolactin can decrease the action of dopamine explaining the worsening of symptoms. An imbalance between the elevation of thyroid hormones and the dopaminergic agonists system may induce RLS in gestation.

Multiparity appears to affect iron levels, potentiating the risk of developing RLS. An imbalance in iron metabolism, transport and storage appears to aggravate the symptoms of this disorder in pregnant women. A decrease in iron and folic acid levels can limit the dopamine synthesis, affecting the pathogenesis of the disease.

Vitamin D has the function of increasing dopamine levels in the brain and also has a protective effect on dopaminergic neurons. A decrease of vitamin D is frequently observed during gestation, which may affect dopaminergic neurotransmission, contributing to an imbalance in dopamine levels.

Dopamine is involved in innumerous processes and a dopaminergic imbalance or dysfunction in the nigrostriatal system may induce the development or worsening of this disorder.

Considering the reviews and studies analyzed, it seems to be possible to associate a common factor related to the affectation of dopamine levels, when variations occur in several hormones (estrogen, progesterone, prolactin, and thyroid hormones), as well as in iron and vitamin D.

In women genetically predisposed to the disorder it appears that pregnancy may trigger its symptomatology. Since not all pregnant women develop RLS during gestation, and the same occurs in other secondary RLS forms, it seems predictable that the predisposition to develop RLS in the idiopathic form and in some of the symptomatic ones might be affected by the genetic background and it appears to have a crucial role.

Although some allelic variants predisposing to idiopathic RLS (loci: MEIS1, BTBD9, and MAP2K5) are already known, it is important to perform further investigations that analyze the frequency of these variants in a large scale of women with this “secondary” pregnancy-related RLS form. It is necessary to identify and connect to gestation, a specific genetic predisposition capable of triggering the phenotype of RLS.

Zinc and magnesium deficiency may occur during pregnancy, and it appears to have a connection between these two elements and RLS.

Several perspectives have been presented, creating the need for additional and thorough research.
